# The impact of nursing interventions on the rehabilitation outcome of patients after lumbar spine surgery

**DOI:** 10.1186/s12891-024-07419-9

**Published:** 2024-05-04

**Authors:** Jun Liang, Liyan Wang, Jialu Song, Yu Zhao, Keyan Zhang, Xia Zhang, Cailing Hu, Dong Tian

**Affiliations:** 1 Department of Orthopaedic Surgery, Shanxi Bethune Hospital,Shanxi Academy of Medical Sciences, Tongji Shanxi Hospital, Third Hospital of Shanxi Medical University, No. 99, Longcheng Street, Taiyuan city, Shanxi Province 030032 China; 2 Tongji Hospital, Tongji Medical College, Huazhong University of Science and Technology, Wuhan, 430030 China

**Keywords:** Lumbar spinal stenosis, Lumbar fusion surgery, Nursing interventions, Postoperative recovery, Pain management, Inflammatory markers

## Abstract

**Background:**

This study aimed to investigate the impact of nursing interventions on the rehabilitation outcomes of patients after lumbar spine surgery and to provide effective references for future postoperative care for patients undergoing lumbar spine surgery.

**Methods:**

The study included two groups: a control group receiving routine care and an observation group receiving additional comprehensive nursing care. The comprehensive care encompassed postoperative rehabilitation, pain, psychological, dietary management, and discharge planning. The Visual Analogue Scale (VAS), Oswestry Disability Index (ODI), Short-Form 36 (SF-36) Health Survey, self-rating depression scale (SDS) and self-rating anxiety scale(SAS) were used to assess physiological and psychological recovery. Blood albumin, haemoglobin, neutrophil counts, white blood cell counts, red blood cell counts, inflammatory markers (IL-6, IL-10, and IFN-γ) were measured, and the incidence of postoperative adverse reactions was also recorded.

**Results:**

Patients in the observation group exhibited significantly improved VAS, ODI, SF-36, SDS and SAS scores assessments post-intervention compared to the control group (*P* < 0.05). Moreover, levels of IL-6, IL-10, and IFN-γ were more favorable in the observation group post-intervention (*P* < 0.05), indicating a reduction in inflammatory response. There was no significant difference in the incidence of postoperative adverse reactions between the groups (*P* > 0.05), suggesting that the comprehensive nursing interventions did not increase the risk of adverse effects.

**Conclusion:**

Comprehensive nursing interventions have a significant impact on the postoperative recovery outcomes of patients with LSS, alleviating pain, reducing inflammation levels, and improving the overall quality of patient recovery without increasing the patient burden. Therefore, in clinical practice, it is important to focus on comprehensive nursing interventions for patients with LSS to improve their recovery outcomes and quality of life.

## Introduction

Lumbar spinal stenosis (LSS) is a common spinal disease in clinical orthopaedics that is characterized by narrowing of the spinal canal, leading to compression of the nerve roots and spinal cord and resulting in a series of symptoms [1]. LSS is considered a developmental disease that has a weak association with age, although the incidence of LSS in individuals older than 60 years is quite high [2]. LSS is prone to recurrence and clinically manifests with symptoms such as low back pain, sciatica, and lower limb weakness [3]. Severe cases can lead to urinary incontinence, muscle atrophy, and difficulty walking. Currently, lumbar fusion surgery is the main treatment method for lumbar spinal diseases, including lumbar spinal stenosis, although minimally invasive decompression surgery is becoming increasingly common. Compared to traditional surgical methods, lumbar fusion surgery has the advantages of better stability, preservation of intervertebral disc function, and reduced risk of complications [4]. Therefore, postoperative nursing interventions for this disease have become a new topic of research interest [5–7]. This study aimed to investigate the impact of nursing interventions on the rehabilitation outcomes of patients after lumbar spine surgery and to provide effective references for future postoperative care for patients undergoing lumbar spine surgery.

## Materials and methods

### Basic information

Eighty patients with LSS treated at Shanxi Bethune Hospital of Shanxi Medical Academy from January 2023 to November 2023 were selected. The Shanxi Bethune Hospital’s institutional ethical review board approved this study, and all patients’ families provided written informed consent. All patients underwent lumbar fusion surgery. Using the random number table method, the patients were divided into a control group and an observation group, with 40 patients in each group. Both the control group and the observation group included 21 males and 19 females. There were no significant differences between the two groups in terms of age, sex, height, weight, American Society of Anesthesiologists (ASA) classification, or other data (Table 1).

The inclusion criteria were as follows:

- Met the diagnostic criteria for lumbar spine diseases according to the “Clinical Diagnosis and Treatment Guidelines for Orthopedics [8]”.
- Age between 18 and 65 years, ASA Grade I to II, required lumbar spine surgery based on their diagnosis.
- No significant abnormalities in the results of preoperative routine blood tests or liver and kidney function tests.
- No contraindications for anaesthesia or surgery.

The exclusion criteria were as follows:

- Patients with significant organ dysfunction.
- Patients with infectious diseases.
- Patients with neurological or psychiatric disorders.
- Patients who were allergic to the drugs used for anaesthesia.
- Patients with severe cerebrovascular disease, heart disease, increased intracranial pressure, or elevated eye pressure.

### Nursing methods

The control group of 40 patients received routine nursing care, which included advising the patients’ families about the process and timeline of postoperative recovery; providing appropriate pain relief medication under the guidance of a doctor according to the patients’ level of pain to alleviate postoperative pain [9]; guiding patients to gradually start activities such as getting out of bed and walking on the second day after surgery but avoiding strenuous activities and heavy lifting; and offering appropriate dietary guidance according to the patient’s condition, such as a low-fat, high-protein, high-fibre diet. The nurses of the control group patients recorded and regularly organized the patients’ daily data according to the study protocol.

In addition to routine care, patients in the observation group received comprehensive nursing care, which included postoperative pain management [10], postoperative exercise management, postoperative emotional management, and postoperative dietary management. Each patient was assigned to a dedicated nurse who recorded detailed daily data, including daily physical recovery status. The specific nursing content is described below.

- Postoperative Rehabilitation Management.

After lumbar spine surgery, patients may undergo a period of rehabilitation. Lumbar spine surgery can cause physical and psychological burdens and stress to patients, and they are likely to face complications, such as deep vein thrombosis and pneumonia. Rehabilitation management can reduce the occurrence of complications through appropriate position adjustments, breathing training, bed transfer, and mobility training [11].

Before starting rehabilitation training, nurses provided guidance to patients on postoperative precautions, correct postures, position adjustments, avoidance of overexertion and incorrect movements. From the third day after surgery until discharge, patients were assisted by nurses to walk before breakfast, lunch, and dinner for 10 min each time, three times a day; the specifics were adjusted based on the patient’s situation [12]. Beginning on the fifth day after surgery, patients performed supine sit-ups with the nurses’ assistance. Patients placed their hands at their sides, and then nurses gently lifted the patients’ hips, forming a bridge shape with the body, which was held for 1–3 s before lowering; this exercise was repeated 5–10 times. Beginning on the seventh day after surgery, patients performed side-lying leg lifts with the nurses’ assistance. The patient lay on one side, supporting their head with one hand and placing the other hand in front for support, as nurses gently lifted the patient’s waist, lifted the upper leg as high as possible, and slowly lowered it [13] ; this exercise was repeated 10 times for each side. Two weeks after surgery, patients performed sit-ups with the nurses’ assistance, lying supine with their feet flat on the bed and their hands crossed over the chest or behind the ears. Then, the nurses gently lifted the patient’s waist, using the abdominal muscles to lift the upper body forwards as close to the knees as possible, and then slowly lowered it; this exercise was repeated 10–15 times. Nurses adjusted the exercises according to the patient’s condition and tolerance and taught them the correct posture, position, and movement techniques to help them avoid overexertion and incorrect movements, reducing the risk of further injury or damage to the lumbar spine [14].

b. Postoperative Pain Management.

According to medical orders, nurses used pain relief techniques, such as cold compresses, hot compresses, massage, and electrotherapy, to alleviate patients’ pain. Nurses regularly observed patients’ pain conditions and record detailed information about pain characteristics, intensity, duration, etc., so that doctors could adjust the pain relief treatment plan. In addition, nurses provided education and guidance to patients and their families about postoperative pain management, including how to correctly use pain relief medications, how to apply pain relief techniques, and how to observe and record pain conditions.

c. Postoperative Psychological Management.

After lumbar fusion surgery, patients may face uncertainties in postoperative recovery and worries about the results of the surgery, which may trigger anxiety and fear. Additionally, after lumbar fusion surgery, patients may experience changes in quality of life, such as dependence on others, limited mobility, and work impact, which can negatively affect patients’ psychological state and self-esteem [15]. Therefore, nurses provided psychological interventions and resources to help patients cope with and adapt to the surgery and recovery process.

Nurses assessed patients’ psychological states through interviews and observations, determining whether patients exhibit anxiety, fear, depression, or other psychological issues. Nurses provided patients and their families with detailed information and education about the surgery and recovery process, which helped them understand the purpose, process, and expected effects of the surgery, alleviating anxiety and fear, and listened to patients’ needs and emotional expressions to provide emotional support and comfort. Nurses used different psychological intervention techniques, such as cognitive-behavioural therapy, relaxation training, and mindfulness exercises, to help patients adjust their negative thoughts and emotions and improve their coping abilities; nurses also regularly followed up with patients to understand changes in their psychological state and recovery progress, and promptly identify and address psychological issues. Moreover, nurses collaborated with mental health professionals to provide necessary counselling and treatment resources to help patients deal with psychological problems [16].

d. Postoperative Dietary Management.

After lumbar spine surgery, patients’ wounds need to heal, and they face issues such as limited mobility and constipation. Therefore, good dietary management can provide sufficient nutrients to promote wound healing and tissue repair, improving patients’ recovery outcomes and quality of life [17].

Before managing the postoperative diet, the nursing staff conducted a comprehensive assessment of patients, including understanding their dietary preferences, food allergy history, nutritional status, and oral health status, to develop a personalized dietary management plan. Based on the assessment results, nursing staff provided a nutritionally balanced diet that included sufficient protein, vitamins, minerals, and carbohydrates to meet patients’ energy needs and promote wound healing. The nursing staff provided small and frequent meals, controlling the quantity and frequency of food intake to avoid overeating, which can lead to weight gain and indigestion. In postoperative dietary management, nursing staff provided easily digestible food, including low-fat and low-fibre foods such as cooked vegetables, tenderly cooked meat, and fish, to reduce the gastrointestinal burden. Patients’ weight changes, nutritional intake, digestive issues, etc., were closely observed so that the dietary management plan could be adjusted in a timely manner.

e. Postoperative Discharge Management.

Nurses provided detailed postdischarge guidance to patients the day before discharge, including precautions during the postoperative recovery period, dietary adjustments, and medication management, so that patients and their families clearly understood the postoperative precautions and could manage themselves and recover correctly. Nurses reminded patients to rest, allocate time for activities and work reasonably and avoid long periods of standing that could burden the waist. In addition, nurses maintained constant contact with patients through interviews and phone calls and recorded patients’ daily vital signs and lumbar spine recovery status within one month after discharge.

Nurses also emphasized to patients the prohibition on bending, carrying, or lifting heavy objects. Patients were told to avoid engaging in high-intensity and heavy labour to prevent damage to the postoperative lumbar spine. Nurses provided positive psychological support and encouragement to patients after discharge, helping them adjust their emotions and strengthen their confidence in recovery. They also reminded patients to stay warm and avoid overexerting the waist.

### Observation indicators

The visual analogue scale (VAS) was used to assess pain before and 1 month after surgery in both groups of patients; on this scale, 0 is no pain, 1–3 is mild pain, 4–6 is moderate pain, and 7–10 is severe pain [18]. The Oswestry Disability Index (ODI) was used to evaluate functional recovery before and 1 month after surgery in both groups of patients [19] ; the ODI includes items on self-care ability in daily life (washing, dressing, etc.), ability to lift heavy objects, walking, sexual life, social activities, and travel (outings). Each item has 6 possible answers, scored from 0 to 5, with 0 indicating no pain and 5 indicating extreme pain and the most severe disability. The scoring method was as follows: actual score/50 (highest possible score) × 100%. If one question was not answered, then the scoring method was calculated as follows: actual score/45 (highest possible score) × 100%. Higher scores indicated more severe functional impairment. The Short-Form 36 (SF-36) Health Survey was administered before and 1 month after surgery in both groups of patients. The SF-36 is a brief self-administered questionnaire that generates scores across 8 dimensions of health: physical functioning (PF; 10 items), general health (GH; 5 items), role limitations due to physical health problems (role physical, RP; 4 items), bodily pain (BP; 2 items), social functioning (SF; 2 items), vitality (VT; 4 items), role limitations due to emotional problems (role emotional, RE; 3 items), and mental health (MH; 5 items). For each domain, a score ranging from 0 to 100 was assigned, with a higher score indicating better health [20]. Self-rating depression scale (SDS) scores were determined before and 1 month after surgery in both groups of patients. The SDS contains 20 items that reflect subjective feelings of depression. Answers are rated on a 4-point Likert scale (from 1, “no or a little of the time,” to 4, “most of the time or all the time”), and the scale includes 10 symptom-positive items and 10 symptom-negative items [21]. Self-rating anxiety scale (SAS) scores were determined before and 1 month after surgery in both groups of patients. The SAS is also composed of 20 items and is rated on a 4-point Likert scale (from 1, “no or a little of the time,” to 4, “most of the time or all the time”). Higher scores reflect more severe anxiety symptoms [22]. Blood albumin, red blood cell counts and haemoglobin levels were compared before surgery, 1 day and 1 month after surgery in both groups of patients; blood was sampled using vacuum methods and analysed using the bromocresol green and cyanide methods, respectively. Neutrophil counts and white blood cell counts, were compared before and 1 month after surgery in both groups of patients; blood was sampled using vacuum methods and analysed using an automatic blood cell analyser. IL-6, IL-10, and IFN-γ levels were compared before and 1 month after surgery in both groups of patients; blood was sampled using vacuum methods and analysed using flow cytometry. Blood samples were collected at the same time as those for routine blood tests and did not require additional invasive procedures. Statistics on the incidence of adverse reactions within 48 h after surgery were calculated in both groups of patients; the main adverse reactions included pressure ulcers, pulmonary infection, venous thrombosis (determined by ultrasound), urinary system infection, neutrophilia (more than 7.5 × 10^9^/L), and leucocytosis (more than 10 × 10^9^/L). The occurrence of delayed wound healing was assessed in both groups.

### Statistical analysis

Count data are expressed as a percentage and were analysed using the χ2 test. The measurement data are expressed as the mean value ± standard deviation. Paired t tests were used to compare the results before and after the intervention. Independent samples t tests were used to compare the results between the two groups except red blood cells, haemoglobin and blood albumin, for the three outcomes, analysis of variance was conducted. A P value less than 0.05 was considered to indicate statistical significance.

## Results

### Comparison of general information between the two groups of patients

There were no statistically significant differences between the two groups of patients in terms of sex, age, height, weight, ASA I or ASA II (*P* > 0.05) (Table 1).

**Table 1:** Comparison of general information between the two groups of patients

	Control group (*n* = 40)	Observation group (*n* = 40)	P
Sex male (female)	21(19)	21(19)	1
Age	53.30 ± 10.67	53.43 ± 11.29	0.96
BMI(kg/m^2^)	24.56 ± 3.75	25.42 ± 3.97	0.33
ASA I (II)	22(18)	27(13)	0.25

### VAS and ODI scores

Table 2 shows the VAS and ODI scores, which indicated that compared with the preoperative conditions, both nursing strategies clearly lowered the VAS-leg score, VAS-back score and ODI 1 month after surgery (*p* < 0.05). Compared with those in the control group, the decreases in scores in the observation group were significantly greater.

**Table 2:** Comparison of the VAS and ODI scores between the two groups

Grouping	n	Time	VAS-leg	P	VAS-back	P	ODI	P
Control group	40	Preoperative	5.90 ± 2.31		4.53 ± 2.42		51.04 ± 16.71	
		Postoperative	1.68 ± 1.52	0.001	1.80 ± 1.89	0.001	33.01 ± 15.65	0.001
		Postoperative-Preoperative	-4.23 ± 1.981		-2.73 ± 2.264		-18.037 ± 17.955	
Observation group	40	Preoperative	6.55 ± 1.76		5.88 ± 2.78		58.98 ± 21.82	
		Postoperative	1.18 ± 1.29	0.001	1.65 ± 1.05	0.001	27.03 ± 9.29	0.001
		Postoperative-Preoperative	-5.38 ± 2.022	0.012	-4.23 ± 3.401	0.023	-31.951 ± 24.994	0.005

### SF-36, SAS and SDS results

Table 3 shows the results of the SF-36, SAS and SDS, which indicated that compared with the preoperative conditions, both nursing strategies clearly lowered the SAS and SDS scores and increased the scores of all 8 domains of the SF-36 1 month after surgery (*p* < 0.05). Compared with those of the control group, the decrease in the SAS and SDS scores for the observation group was significantly greater, and the increase in the scores for 6 domains of the SF-36, except the RE and MH domains, for the observation group were obviously greater.

**Table 3:** Comparison of the SF-36, SAS and SDS scores between the two groups

Grouping	Control group			Observation group		
n	40			40		
Time	Preoperative	Postoperative	Postoperative-Preoperative	Preoperative	Postoperative	Postoperative-Preoperative
SAS	53.63 ± 9.60	42.15 ± 10.08	-11.475 ± 10.063	51.68 ± 9.53	46.03 ± 10.05	-5.651 ± 8.341
*P*		0.001			0.001	0.006
SDS	55.93 ± 10.11	42.65 ± 7.91	-13.275 ± 13.308	52.78 ± 9.33	45.18 ± 8.35	-7.601 ± 11.913
*P*		0.001			0.001	0.048
PF	46.51 ± 9.55	50.62 ± 8.48	4.125 ± 8.688	46.75 ± 9.97	56.50 ± 11.61	9.750 ± 8.693
*P*		0.005			0.001	0.005
RP	19.37 ± 21.54	46.25 ± 18.38	26.875 ± 20.714	15.00 ± 18.60	51.62 ± 16.38	36.625 ± 16.923
*P*		0.001			0.001	0.024
BP	36.75 ± 13.47	55.01 ± 8.16	18.251 ± 11.958	35.00 ± 9.60	59.00 ± 9.28	24.001 ± 13.165
*P*		0.001			0.001	0.044
GH	47.38 ± 10.12	50.51 ± 7.41	3.125 ± 6.169	45.25 ± 11.65	51.75 ± 6.65	6.501 ± 8.258
*P*		0.003			0.001	0.042
VT	44.88 ± 9.71	54.75 ± 8.76	9.875 ± 8.584	40.63 ± 8.63	56.13 ± 9.23	15.501 ± 10.177
*P*		0.001			0.001	0.009
SF	44.19 ± 14.61	62.81 ± 12.56	18.625 ± 15.790	37.88 ± 16.22	64.19 ± 13.54	26.312 ± 16.860
*P*		0.001			0.001	0.039
RE	21.65 ± 25.65	74.18 ± 21.99	52.522 ± 24.912	57.52 ± 28.23	69.19 ± 23.11	11.672 ± 31.630
*P*		0.001			0.025	0.001
MH	47.21 ± 13.47	74.40 ± 11.05	27.201 ± 16.278	73.30 ± 11.44	79.35 ± 11.49	6.051 ± 13.277
*P*		0.001			0.006	0.001

### Blood test results

Tables 4 and 5 shows the blood test results, which showed that compared with the preoperative conditions or 1 day after surgery, both nursing strategies clearly lowered the IL-6, IL-10 and IFN-γ levels and increased the blood albumin, red blood cell and haemoglobin levels 1 month after surgery (*p* < 0.05). Compared with those in the control group, the decreases in the levels of IL-6, IL-10 and IFN-γ in the observation group were significantly greater, and the increase in the level of blood albumin in the observation group was obviously greater, with the not significant trend for red blood cell count and haemoglobin level.

**Table 4:** Comparison of blood test results 1 between the two groups

Grouping	Control group			Observation group		
n	40			40		
Time	Preoperative	Postoperative	Postoperative-Preoperative	Preoperative	Postoperative	Postoperative-Preoperative
Neutrophil counts(×10^9^/L)	5.96 ± 3.57	6.93 ± 4.26	0.97 ± 4.573	7.49 ± 3.36	6.87 ± 3.05	-0.622 ± 5.091
*P*		0.187			0.445	0.145
White blood cell counts(×10^9^/L)	7.76 ± 2.95	9.08 ± 3.48	1.322 ± 4.021	8.29 ± 3.17	9.54 ± 3.38	1.247 ± 5.506
*P*		0.044			0.160	0.945
*P*		0.001			0.001	0.001
IL-6(pg/ml)	8.02 ± 3.21	4.10 ± 2.39	-1.922 ± 1.726	8.28 ± 3.54	4.71 ± 1.64	-3.573 ± 3.297
*P*		0.001			0.001	0.006
IL-10(pg/ml)	5.92 ± 3.06	4.64 ± 2.15	-1.271 ± 2.163	6.38 ± 2.69	3.85 ± 1.55	-2.528 ± 2.257
*P*		0.001			0.001	0.013
IFN-γ(pg/ml)	3.51 ± 1.36	2.68 ± 0.91	-0.815 ± 1.051	3.92 ± 1.45	2.37 ± 0.61	-1.542 ± 1.207
*P*		0.001			0.001	0.005

**Table 5:** Comparison of blood test results 2 between the two groups

Grouping	Control group				Observation group			
n	40				40			
Time	Preoperative	1 day postoperative	1 month postoperative	1 month Postoperative-1 day postoperative	Preoperative	1 day postoperative	1 month postoperative	1 month Postoperative-1 day postoperative
Red blood cell counts(×10¹²/L)	4.54 ± 0.37	4.05 ± 0.47	4.28 ± 0.44	0.23 ± 0.40	4.49 ± 0.39	4.06 ± 0.52	4.30 ± 0.36	0.24 ± 0.36
*P*			0.001				0.001	0.883
Haemoglobin(g/L)	139.80 ± 14.85	124.65 ± 13.67	130.03 ± 11.54	5.38 ± 10.57	136.68 ± 12.63	122.18 ± 13.65	131.13 ± 11.66	8.95 ± 10.20
*P*			0.001				0.001	0.128
Blood albumin(g/L)	40.96 ± 2.56	35.90 ± 2.96	38.73 ± 2.05	2.83 ± 2.70	40.05 ± 3.34	34.65 ± 3.17	39.12 ± 2.73	4.48 ± 2.76
*P*			0.001				0.001	0.001

### Comparison of adverse reactions occurring within 48 h after surgery between the two groups of patients (%)

There were no significant differences in the occurrence of adverse reactions within 48 h after surgery between the control group and the observation group (*P* > 0.05) (Table 6). In addition, no patients in either group experienced delayed wound healing.

**Table 6:** Comparison of adverse reactions occurring within 48 h after surgery between the two groups of patients

Grouping	n	Time	Pressure ulcer	Pulmonary infection	Venous thrombosis	Urinary tract infection	Neutrophilia	Leukocytosis
Control group	40	Postoperative	1(2.5)	1(2.5)	2(5)	1(2.5)	24(60)	30(75)
Observation group	40	Postoperative	0	0	0	0	19(47.5)	25(62.5)
P			0.314	0.314	0.152	0.314	0.262	0.228

## Discussion

Lumbar fusion can effectively restore normal lumbar function in LSS patients, but the necessity of postoperative bed rest causes various types of psychological and physical discomfort to patients. Therefore, postoperative nursing care interventions are crucial for patient recovery [23]. In this study, the observation group received comprehensive nursing care interventions based on routine care for LSS, including postoperative rehabilitation management, postoperative pain management, postoperative psychological management, postoperative dietary management, and postoperative discharge management, which offered nursing interventions from psychological and physiological perspectives to help alleviate pain and improve postoperative recovery outcomes in patients. This approach provides thorough and personalized nursing to patients. Although some studies have suggested that after lumbar decompression, physical therapy intervention does not significantly affect clinical outcomes, as measured by patient‑reported outcomes and surgical outcomes [24, 25], numerous approaches aimed at improving nursing quality, such as brain storming, world café, and management by objectives, have been created since the World Health Organization has defined quality nursing as a patient-oriented, fair, convenient, effective, highly efficient, safe and acceptable model of nursing [26]. Studies have shown that personalized nursing that involves the application of a scientific, systemic and standardized nursing program and plan is effective for postoperative rehabilitation [27, 28]. In addition, intimate and comprehensive nursing also contributes to communication and relationships between patients and nurses, which is beneficial for improving the quality of nursing and the outcome of surgery [29–31].

The results also supported the efficacy of comprehensive nursing. After receiving comprehensive nursing interventions, patients in the observation group had better scores on the VAS, ODI, SAS, SDS, and 6 of the 8 domains of the SF-36 than did patients in the control group (*P* < 0.05). The study results indicate that comprehensive nursing care interventions based on routine care can significantly impact patients’ postoperative psychological and physiological conditions, enhancing postoperative recovery outcomes. Blood albumin, red blood cell count and haemoglobin are three common outcomes used to assess nutrient conditions, and our results suggested no significant results for the later two outcomes; however, both nursing methods clearly improved nutrient conditions compared with preoperative conditions, which suggested a positive effect of the comprehensive nursing method. The differences in neutrophil and white blood cell counts between the two groups were not clear; however, we used more microscopic outcomes to compare inflammatory conditions. The measurement of cytokine levels is important for predicting postoperative complications and inflammation severity. For example, increases in IL-6 and IFN-γ levels are associated with sepsis and wound disruption [32, 33], while the IL-10 concentration can be used to determine the occurrence of postoperative complications such as atrial fibrillation [34]. In this study, after nursing interventions, the levels of IL-6, IL-10, and IFN-γ in the observation group were improved compared with those in the control group (*P* < 0.05), suggesting that comprehensive nursing interventions, especially in dietary management and pain management, can reduce patients’ levels of inflammatory factors and improve patient recovery. However, both groups received non-steroid anti-inflammatory drug, which may influence the results. The lack of delayed wound healing in patients also suggested the high efficacy of comprehensive nursing. In addition, a comparison of the incidence of adverse reactions within 48 h after surgery in both groups found no significant difference between the two groups after receiving nursing interventions (*P* > 0.05), indicating that not only did comprehensive nursing interventions improve several positive health indicators, but they also did not negatively impact the risk of postoperative adverse reactions in patients.

There were two major limitations in our study. First, the number of included patients was small, and the follow-up duration was short, which may influence the results. Second, there are many perioperative factors that may all influence the different outcomes in the two groups; we investigated only the nursing field, which may cause bias.

## Conclusion

In summary, this study demonstrated that comprehensive nursing interventions have a significant impact on the postoperative recovery outcomes of patients with LSS, alleviating pain, reducing inflammation levels, and improving the overall quality of patient recovery without increasing the patient burden. Therefore, in clinical practice, it is important to focus on comprehensive nursing interventions for patients with LSS to improve their recovery outcomes and quality of life. Further studies with more included patients are needed to verify our results.

## Data Availability

All data generated or analysed during this study are included in this published article.

## Abbreviations

ASA: American Society of Anesthesiologists
BP: bodily pain
GH: general health
LSS: lumbar spinal stenosis
MH: and mental health
ODI: Oswestry Disability Index
PF: physical functioning
RP: role physical
SAS: self-rating anxiety scale
SDS: self-rating depression scale
SF: social functioning
SF-36: Short-Form 36
VAS: visual analogue scale
VT: vitality
RE: role emotional
